# Genomic Characterization of Quality Wool Traits in Spanish Merino Sheep

**DOI:** 10.3390/genes15060795

**Published:** 2024-06-17

**Authors:** Gabriel Anaya, Nora Laseca, Antonio Granero, Chiraz Ziadi, Francisco Arrebola, Andrés Domingo, Antonio Molina

**Affiliations:** 1 MERAGEM Research Group, Department of Genetics, University of Córdoba, CN IV KM 396, 17071 Córdoba, Spain; b22ancag@uco.es (G.A.); z72zizic@uco.es (C.Z.); 2 National Association of Merino Sheep Breeders (ACME), 28007 Madrid, Spain; 3 Agriculture, Livestock and Fisheries Research Institute (IFAPA), 14270 Cordoba, Spain; 4 Center of Selection and Reproduction Animals (CENSYRA), 06007 Badajoz, Spain

**Keywords:** Merino sheep, GWAS, NGS, wool traits, fine wool

## Abstract

The native Spanish Merino breed was the founder of all the other Merino and Merino-derived breeds worldwide. Despite the fact that this breed was created and improved to produce the highest quality fine wool, the global wool market crisis led to the wholescale crossing of most of the herds with breeds for meat purposes. Nevertheless, there are still some purebred animals with a high potential for producing quality wool. The objective of this study was to characterize the current wool quality of the breed and identify genes associated with these parameters. To achieve this, over 12,800 records from the most representative animals of the breed (registered in the herd book) were analyzed using the Australian OFDA 2000 system, for parameters such as fiber diameter (FD), standard deviation (SD), coefficient of variation (CV), fibers over 15 microns (>15%), staple length (SL), and comfort factor (CRV). Additionally, animals with the most extreme FD values were whole-genome sequenced using NGS. Genome-wide association studies (GWAS) determined the association of 74 variants with the different traits studied, which were located in 70 different genes. Of these genes, *EDN2*, *COL18A1*, and *LRP1B,* associated with fibers over 15%, and *FGF12* and *ADAM17*, associated with SL, play a key role in hair follicle growth and development. Our study reveals the great potential for recovering this breed for fine wool production, and identifies five candidate genes whose understanding may aid in that selection process.

## 1. Introduction

Genomic studies have confirmed that the Spanish Merino sheep is the origin of all other Merino and Merino-derived breeds worldwide [1,2]. The origin of the breed dates back to Roman times in the Iberian Peninsula, through the improvement of native sheep to obtain animals with a white fleece and fine wool, showing that, from the very beginning, the breed was focused on producing high-quality wool [3].

For centuries, the Spanish Merino population was not affected by any external influences, and its special wool purpose was kept up until the middle of the 20th century when, due to the world wool crisis, many breeders crossed their sheep with other foreign breeds with greater meat production such as Merino Precoce, Fleischschaf, Landschaf, or Île de France, among others, in order to produce animals with this dual use, like the Australian Merino [4,5]. The increase in meat production through this crossbreeding process led to a decrease in the quality of wool in the population, leading to the eventual disappearance of some herds whose sole objective was wool production [6].

To date, only a few breeders have retained practically uncrossed herds, which show the morpho-structural and genomic characteristic of the ancestral population, and therefore its excellent wool quality. Some of these herds have been maintained through closed inbreeding for many generations, resulting in the formation of well-defined genetic lines, which can be clearly distinguished from the undifferentiated animals with phenotypes closer to Merino (Merino type) [7,8,9,10].

For these reasons, there are currently about 3.2 million Spanish Merino-type sheep, of which only 130,000 individuals are registered as purebred in the breed register by the National Association of Merino Sheep Breeders (ACME) [11]. Due to the high number of crossbred ewes of different degrees, wool quality varies greatly between herds and only those herds that have remained purebred retain homogeneously high levels of quality. One common practice consists of blending high-quality wool sourced from purebred Merino sheep with standard-quality wool from other crossbred Spanish sheep, resulting in a devaluation of these superior fleeces and a subsequent decrease in wool prices. Furthermore, the Spanish Merino production system remains largely extensive, with animals raised in mountain pastures or in the unique Iberian habitat known as the “Dehesa”, under semi-free conditions. Consequently, the promotion of wool quality as a premium product is intricately linked to the preservation of the pasture and/or mountain ecosystems in which the animals are raised.

Since 2016, the National Association of Merino Sheep Breeders has promoted the revaluation of wool by incorporating it as an objective in the breed’s improvement plan. In this context, a wool quality control organization was set up, in which about 7000 fleeces are analyzed per year. However, the aim is to go further, and actively search for DNA variations located within or near genes used in marker-assisted selection. Therefore, although genomic tools have already been used to search for genes related to wool quality in some breeds, this is, to the best of our knowledge, the first work carried out in the pure Spanish Merino breed. In addition, our study is the first genomic study in this breed to use NGS approaches. To achieve this, low coverage NGS at a depth of 4x was shown to be a useful, accurate, and reliable tool for detecting polymorphic sites, inferring genotypes [12], or conducting population assays [13].

The aims of the present work were therefore to (i) characterize the current wool traits of pure Spanish Merino sheep; and (ii) search for genes related to the wool quality parameters studied.

## 2. Materials and Methods

### 2.1. Ethical Statement

This study did not require at any time the usual handling/management of animals, since we worked directly with the records provided by the National Association of Merino Sheep Breeders. Likewise, the blood samples used were those collected in 2022 during routine and mandatory health controls by the relevant governmental administrations, and taken by official veterinarians in full compliance with animal welfare standards.

### 2.2. Animal Selection and Phenotyping

A total of 559 sheep of the Spanish Merino breed were sampled for wool quality traits during the years 2022 and 2023. All the animals analyzed came from two herds kept at official centers which receive animals from all the herds that breed pure animals, with all the variability of the breed concentrated in these two herds: the Centre of Animal Selection and Reproduction (CENSYRA, Badajoz, Spain), where a cohort of females was raised to test Merino rams; and the Andalusian Institute of Agriculture, Fisheries, Food and Ecological Production Research and Training. (IFAPA, Andalucía, Spain), which keeps breeding females from all the recognized lines of this breed. The wool samples were obtained from the area between the back of the neck and the top of the scapulae, and were analyzed in the CENSYRA, where the fiber diameter (FD), standard deviation (SD), coefficient of variation (CV%), comfort factor (CF%), percent of fibers less than 15 microns (<15%), staple length (SL) and fiber curvature (CRV) were measured with the Australian OFDA 2000 (BSC Electronics, Ardross, Australia). The description of each of the traits is reflected in Table 1. The descriptive statistic of the traits was calculated for the entire population and by sex with the IBM SPSS Statistics 25.0 software for Windows (IBM Corp., Armonk, NY, USA). The differences between the values of each trait based on sex were calculated using a mean comparison test.

### 2.3. Sequencing, Alignment and Quality Control

The 76 animals with the highest and lowest values were sequenced according to the fiber diameter into two different groups (42 with high wool quality formed by those with the lowest fiber diameter and 34 with low wool quality represented by animals with the highest fiber diameters). Blood samples from the selected individuals were collected by jugular venipuncture in vacutainers, with EDTA K3 as the anticoagulant. Genomic DNA was purified with the DNeasy Blood and Tissue Kit (QIAGEN, Hilden, Germany), following the manufacturer’s instructions. Samples were sent to NEOGEN Genomics (Lincoln, NE, USA). Paired-end sequencing libraries were constructed according to the manufacturer’s instructions (Illumina Inc., San Diego, CA, USA), and then analyzed for size distribution using TapeStation, quantified using Qubit, and sequenced on the Illumina NovaSeq 6000 platform (Illumina Inc., San Diego, CA, USA) at PE150. A total of 617 GB of data was generated. The quality control of the raw data was conducted with fastqc V0.11.9 software (https://www.bioinformatics.babraham.ac.uk/projects/fastqc/ accessed on 24 March 2023). After filtering, the adapters were removed using fastp v0.23.4 software [14]. The remaining high-quality sequences were aligned with the GCA_016772045.1_ARS-UI_Ramb_v2.0 sheep genome (previously indexed), using the Burrows-Wheeler Aligner (BWA) v0.7.18 software with the “-men” command [15]. Next, the bam files generated for each sample were sorted using the “sort” command in Samtools v1.12 [16]. Duplicates were marked and removed using the “MarkDuplicates” and “RemoveDuplicates” commands in Picard v3.1 software (Picard Toolkit. Cambridge, UK, 2018). The samples were then filtered to keep only the reads with paired reads mapped with quality values of 20, and an alignment score above 100, using Samtools v1.12 software [16].

### 2.4. Variant Calling

The genotype likelihood was calculated by adding the allelic depth, genotype depth, and strand bias, using the *Ovis aries* reference genome GCA_016772045.1_ARS-UI_Ramb_v2.0 to generate a bcf file using bcftools (https://github.com/samtools/bcftools accessed on 24 March 2023). Finally, the genotypes were called with bcftools to generate the vcf file.

### 2.5. Genome-Wide Association

To obtain the genome-wide association (GWAS), the vcf file was converted to a binary file using plink v1.9 software [17]. The resulting file contained 41,093,307 DNA variants distributed over the autosomal chromosomes OAR1 to OAR26 (coded 1 to 26) and the OARX sexual chromosome (coded as 27). After that, all SNPs in linkage disequilibrium (LD) with a variance inflation factor (VIF) of 2 were pruned to leave 15,734,409 DNA variants, using plink v1.9 [17]. To obtain the GWAS, a Univariable Linear Mixed Model (uvLMM) was employed using GEMMA v0.98.5 software [18], using the following formula:

**y = Wα + xβ + u +** ε

where y is an n-vector of quantitative traits (or binary disease labels) for n individuals; W is an incidence matrix of covariates (fixed effects) including a column of 1 s; α is a vector of the corresponding coefficients including the intercept; x is an n-vector of marker genotypes; β is the effect size of the marker; u is an n-vector of random effects u ∼ N(0, λτ − 1 K), where τ is the variance of the residual errors; λ is the ratio between two variance components; K is the genomic relationship matrix (estimated from the markers); and ε is the n-vector of errors. In addition, the model included a correction for population stratification based on the first ten components of a principal component analysis performed in PLINK v.1.9 as covariates. Data were corrected by population structure, the genomic relationship between individuals, age, sex, and numbers of animals born (first-born, from 2 to 5, 6 and over) and lambs. The assay was repeated for each of the traits studied. Manhattan plots and the quantile–quantile (QQ) plots were obtained using the qqman R package [19] in the R studio environment (RStudio Team 2020 http://www.rstudio.com, accessed on 24 March 2023).

### 2.6. Identification of Candidate Genes

Based on the chromosomal location and relative position according to the GCA_016772045.1_ARS-UI_Ramb_v2.0, the reference genomes of each variant associated with each trait with a *p*-value over 1.0005 × 10^−7^ were tested to find the candidate genes and sorted into 500 Kb windows (250 Kb upstream and 250 Kb downstream from each point) using the BioMart application from the Ensembl repository. Next, the genes were submitted to the DAVID database to study the Gene Ontology.

## 3. Results

### 3.1. Summary Statistics of Phenotype Data

The descriptive statistics of fiber diameter, standard deviation, coefficient of variation, comfort factor, and staple length are shown in Table 2. Fiber diameter ranged from 15.2 to 28.5 microns, with an average of 22.2 microns. The SD mean was 3.9 microns, with an average CV% of 17.5%. Almost the entire population showed a high CF% value, with a mean of 95.7%. The mean of fibers less than 15 microns per sample was 2.7%, and ranged from 0 to 44.4%, including the highest CV (131%). The SL ranged from 15 to 85 mm, with an average of 42.6. Finally, the mean curvature of the fiber was 119.14 degrees per mm. The mean comparison test showed that there was a significant difference in all the studied traits between males and females.

### 3.2. Statistic of Sequencing Data

The sequencing process generated an average of 15.97 Gb data of information for each sample, with a median coverage of 5.32× and over 105 million reads. The reads were of a high quality, with an average Q20 of 97.17% and a Q30 of 92.58% before filtering. The mean values of Q20 and Q30 after filtering were 97.66% and 93.18%, respectively, with 17.17% of the regions duplicated.

### 3.3. Genome-Wide Association and Identification of Candidate Genes

**Figure 1:**
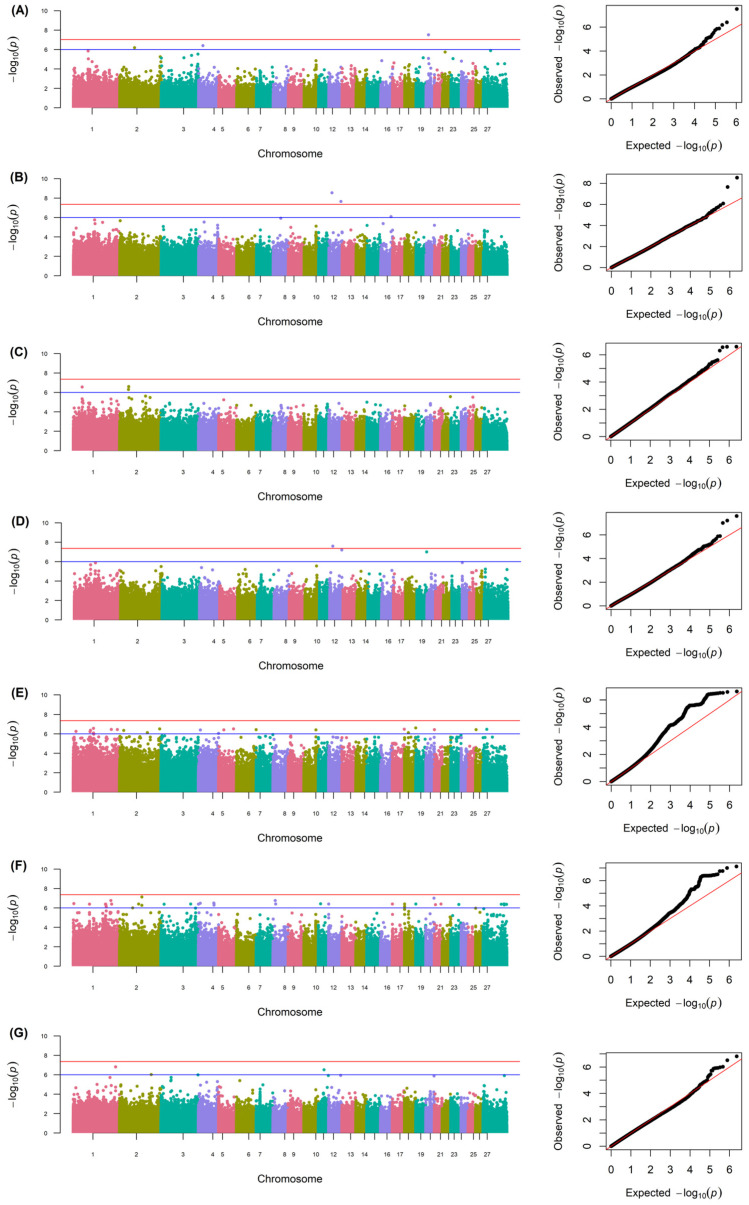
Manhattan plots and quantile–quantile (QQ) plots of the associated DNA points from genome-wide association of fiber diameter (**A**), standard deviation (**B**), coefficient of variation (**C**), comfort factor (**D**), fibers under 15 microns (**E**), staple length (**F**), and fiber curvature (**G**).

Figure 1 shows the Manhattan plots for the 7 traits studied. The Univariable Linear Mixed Model showed a total of 74 SNPs associated with wool quality traits located in the *Ovis aries* chromosomes (OAR1, 2, 3, 4, 5, 6, 8, 10, 11, 12, 16, 17, 18, 19, 20, 21, 23, 26, and X). Of these, 3 were associated with FD, 3 with SD, 4 with CV, 3 with CF, 23 with <15%, 35 with SL, and 3 with CRV (Table 3). A total of 70 different genes were located within or near the 74 variants detected. A total of 42 of these SNPs were located within 41 different genes, while the other 22 were close to different candidate genes in a range, with the closest one being the *FANCG* gene related to CV (331 bp upstream from the SNP), and the furthest gene, *MKRN3*, associated with SL (303,877 bp upstream from the SNP). A total of 27 of the genes were not officially named. The most significative SNPs were within the *USH2A* gene located in the OAR12 and associated with SD (2.880708 × 10^−9^). Some of the SNPs associated with a specific trait were within (*RCAN1* for the fibers under 15 microns) or near the same gene (*MKRN3* for the trait of staple length). Two SNPs (OAR12:18454843 and OAR12:72689402, found within the *USH2A* and *SYT14* genes, respectively) were associated with both SD and CF traits.

## 4. Discussion

The results of the present work show that the wool of the pure Spanish Merino continues to be among the finest, despite the fact that for decades, wool quality has not been used as a criterion for selection, with the fiber diameter equaling or surpassing that of other breeds previously studied, such as Akkraman [20], Chinese Merino [21,22,23,24], Crossed Merino [25], or Rambouillet [26]. However, the diameter of the fibers currently observed in Spanish Merino appears to be significantly coarser compared to those typically found in breeds highly selected for producing fine wool, such as Uruguayan sheep, which boast a remarkable fineness, with fibers measuring around 16.6 microns [27], or the Australian Merino, with an average fiber diameter below 16.5 microns [28]. In this way, the highly specific selection being carried out in these breeds can serve as a model for its application in the Spanish Merino to reduce the FD.

Several of the wool quality parameters in Spanish Merino have been studied previously [29], and in recent years, the ACME association has focused on improving wool quality. Although the current average fiber diameter is 1 micron greater than 15 years ago, there are animals with average values of 15.2 microns, which represents a much greater fineness than that found 15 years ago (min: 18 microns) and very close to the figures for breeds which are ultra-selected for wool fineness [27,28]. We can therefore confirm the presence of a foundation stock of animals in the breed with ultra-fine wool, as evidenced by the percentage of fibers measuring below 15 microns. While the population average stands at 2.74% per individual, some animals exhibit as much as 44% of fibers below 15 microns. The comfort factor has improved considerably over the last decade from an average of 66.2% to 95.66%, while regarding fiber length, the coefficient of variation has increased, reflecting that the breed remains highly heterogeneous.

One of the parameters that has decreased is fiber length, which is currently about 2 cm shorter with significant variation among individuals compared to values recorded 20 years earlier [29,30]. This is mainly because breeders who have begun to prioritize the production of high-quality wool still maintain dual-purpose breeding (for meat and wool), noticing that females giving birth more than once a year or experiencing multiple pregnancies exhibit a lower wool growth due to the high demand for energy required by both processes [31]. Compared to other fine-wool-producing breeds, the fiber length of the Spanish Merino is shorter [22], and so staple length (SL) is an aspect that needs to be improved in the breed, in line with industry standards. For all these reasons, more importance is currently given to this trait in the genetic evaluations carried by the National Association of Merino Sheep Breeders. A point to consider is that the fiber of the males’ wool is coarser, and their fleece contains less ultrafine fiber compared to that of the females, although the fiber length of the males is longer (Table 2). This is to be expected due to the pronounced sexual dimorphism present in the breed.

However, although Merino wool has shorter fibers, it presents other characteristics that make it unique, such as the size of the fleece, which is larger than that found in other breeds, the high density of fibers, and especially the curvature of the fiber, which is an interesting property with a view to producing specialized clothing.

Association studies have proven to be a valuable tool in the search for candidate genes related to various traits in sheep, by identifying fixed alleles among the groups analyzed [20,21,22,27]. The relationships between individuals and the structure of the population can lead to the appearance of false positives in these types of association studies [32,33], and in our GWAS, a linear mixed model corrected by population stratification and individual relationship was employed to avoid them. QQ plots support the reliability of the model used and the results obtained (Figure 1). The gene exploration was conducted based on proximity to the identified variant, by which all those within a specified window size upstream or downstream of the sequence are proposed, assuming they are within the same linkage blocks. In some cases, the variant is located directly in the intronic or exonic region of a specific gene.

In our study, 10 genes were proposed as candidates for traits related to fiber diameter and their level of homogeneity in the sample and/or population (FD, SD, and CV). Several of these, such as *CTNND2*, *LRFN2*, or *SYT14*, encode membrane proteins and play important roles in cell adhesion, although none play a known role in wool generation. Similarly, none of the genes associated with the comfort factor appear to be directly related to follicular development or wool generation, although this may be due to our limited knowledge of the ovine genome.

However, among the candidate genes, five could be related to wool production due to their role in regulating hair follicle growth. Of these, genes *EDN2*, *COL18A1*, and *LRP1B* were associated with the percentage of fibers over 15 microns. Gene *EDN2*, located on OAR1, synthesizes endothelin, a cytokine with a crucial role in the migration of epidermal melanocytes from the hair root, and is produced by keratinocytes [34]. It has also been observed that endothelin, produced by hair follicle progenitors, binds to receptors on dermal sheath cells, triggering their contraction, which is essential for hair follicle regression [35], while gene *COL18A1* (collagen, type XVIII, alpha 1), also located on OAR1, synthesizes collagen protein. Collagen plays a vital role in hair and wool production by maintaining the structure of hair follicles, is responsible for keeping hair in good condition, and is involved in the production of new hair, providing the properties of elasticity and strength. In fact, in humans, a mutation in the *COL18A1* gene leads to severe myopia, together with alopecia areata in the occipital region, indicating its potential importance in the development or health of hair follicles [36]. Gene *LRP1B* (LDL Receptor Related Protein 1B) is located on OAR2. Low-density lipoprotein (LDL) is a cholesterol molecule, and an association has been found between cholesterol levels and hair health. Indeed, the presence of LDL seems to be associated with alopecia and other types of skin and hair disorders [37].

Within the candidate genes associated with fiber length, *FGF12* and *ADAM17*, located on OAR1 and OAR3, respectively, were also proposed. It has been suggested that fibroblast growth factor 12 (*FGF12*) is necessary for the hair growth cycle [38], and it has even been tentatively related to hair length in humans [39]. Meanwhile, another gene belonging to the fibroblast growth factor superfamily (*FGFs*), *FGF5*, was previously associated with SL and greasy wool weight in crossbreed Chinese Merino [40] and crossed Merino [25], while *FGF20* plays a key role in the regulation of the skin compartments during the early morphogenesis of primary wool follicles [41].

In addition, *ADAM17* (disintegrin and metalloproteinase 17) plays a role in skin homeostasis. Specifically, through its effect on keratinocytes, it has been shown that a deficiency of this gene in mice can lead to chronic dermatitis in adulthood [42]. The significant impact it has on keratinocytes suggests that it may play an important role in wool development, as these cells are the basis for wool generation in the follicle [43].

This was the first GWAS carried out in the pure Spanish Merino sheep using a sequencing approach. A number of genes have been proposed as candidates for wool traits in studies of other breeds by [20] (*CEP290*, *PRKCZ*, *TMTC3*, *SLC45A1*), [22] (*LGR4*, *PIK3CA*, *SEMA3C*, *NF1B*, *OPHN1*, *THADA*, *KEGG, UBE2E3*, *RHPN2*), [40] (*FGFs*), [25] (*FGF5*, *STAT3*, *KRT86*, *ALX*), [27] (*IGF1*, *TGFB2R*, *PRKCA*), [24] (*DKK*), and [21] (*YWHAZ*, *KRTCAP3*, *TSPEAR*, *PIK3R4*, *KIFI6B*, *PTPN3*, *GPRC5A*, *DDX47*, *TCF9*, *TPTEL*, *NBEA*). Although we found genes of the same molecular family (*FGFs*) playing an important role in hair development, the lack of agreement with genes proposed in these other studies may be due to differences between breeds and the number of markers used.

However, after detecting the different genes involved in hair growth and development processes, we consider them interesting subjects for potential further investigation. These results require validation using a larger dataset before they can be used in marker-assisted selection in Spanish Merino sheep. Overall, however, our findings will be useful for further genomic studies and genetic improvement programs in this breed.

## 5. Conclusions

In this study, we first characterized Spanish Merino wool though the analysis of seven traits of interest in the fiber industry. Our results show the immense potential of the breed to return to its origins as a producer of high-quality fine wool, with a significant percentage of fine fibers containing many curls. However, the fiber length must be improved to accommodate the demands of the industry. Furthermore, we found up to 74 genome-wide significant SNPs related to these same traits, located within or near 70 genes, and, most importantly, 3 genes associated with fiber percentage less than 15%, and 2 associated with staple length, which play a key role in hair development and/or growth. The new candidate genes are expected to provide a good theoretical basis for optimizing the selection of this breed to obtain high-quality wool. Our study aimed to analyze the situation of the breed as regards wool production, and enhance the current tools for selecting animals with quality wool, focusing on the parameters that require improvement to restore the wool aptitude that made the Spanish Merino the most coveted breed in the world.

## Figures and Tables

**Table 1:** Description of the wool traits analyzed in the present study.

Trait	Code	Description
Fiber Diameter	FD	The average fiber diameter in a staple of wool, measured in microns. Fibers do not have a uniform thickness within the staple because they grow at different rates and speeds. The differences in thickness can be up to 15 microns.
Standard Deviation	SD	Measurement of the variation of wool fiber diameter. Microns of variation between the average of the FD and the minimal and maximal diameter.
Coefficient of Variation	CV	Another measure of variability of the fiber diameter, but expressed as a percentage and relative to the average fiber diameter. This is determined mathematically using the equation: CV% = SD/AFD × 100
Comfort Factor	CF	Percentage of fibers under 30 microns. Fibers wider than 30 microns are rigid and do not bend when they touch the skin, resulting in the wool having a prickly feeling and/or skin irritation.
Fiber under 15 microns	<15%	Percentage of extra fine fibers under 15 microns.
Staple Length	SL	Measurement of the length of the unstretched staple expressed in millimeters.
Fiber Curvature	CRV	Fiber curvature expressed in degrees/millimeters. Generally, a greater curvature is associated with a higher crimp frequency.

Name (Trait), identification (Code) and description (Description) of each of the traits studied in the present work cited in Section 2.2.

**Table 2:** Descriptive statistics of wool traits in the population analyzed.

Trait (Unit)	Code	Group	Mean	Min.	Max.	CV %
Fiber diameter (µm)	FD	Global	22.21	15.2	28.5	10
		Male	25.07	21.4	28.5	7.41
		Female	21.92	15.2	28.2	9.2
Standard deviation (µm)	SD	Global	3.89	2.3	6.6	17
		Male	4.40	2.7	6.4	17
		Female	3.83	2.3	6.6	16.22
Coefficient of variation (%)	CV	Global	17.48	12	29	12
		Male	17.49	12.5	25.2	13.93
		Female	17.48	12	29	11.94
Comfort factor (%)	CF	Global	95.66	70.8	100	6
		Male	87.63	71	100	9.38
		Female	96.46	70.8	100	5.1
Fibers under 15 microns (%)	<15%	Global	2.74	0	44.4	131
		Male	0.55	0	3.6	138
		Female	2.96	0	44.4	125
Staple length (mm)	SL	Global	42.6	15	85	24
		Male	50.78	20	85	28.62
		Female	41.78	15	80	22.58
Fiber curvature (degrees/mm)	CRV	Global	119.14	69.1	171.8	13
		Male	104.81	69.1	140.4	13.31
		Female	120.56	75.1	171.8	12.75

Traits of the present study, FD (fiber diameter), SD (standard deviation), CV% (coefficient of variation), CF% (comfort factor), <15% (percent of fibers under 15 microns), SL (staple length), CRV (fiber curvature) calculated for the entire population (Global) and by sex (Male, Female).

**Table 3:** Genome-wide analysis of significant SNPs for wool production traits.

Trait	Chr	Pos. (bp)	*p*-Value	Nearest Gene	Distance (bp)
FD	2	89,558,512	6.51632 × 10^−7^	*LOC114112820*	−678
	4	25,386,150	3.958888 × 10^−7^	*ENSOARG00020040429*	within
	20	14,330,117	3.015784 × 10^−8^	*LRFN2*	within
SD	12	18,454,843	2.880708 × 10^−9^	*USH2A*	within
		72,689,402	2.22192 × 10^−8^	*SYT14*	within
	16	61,545,925	8.147923 × 10^−7^	*CTNND2*	within
CV	1	52,910,619	2.798289 × 10^−7^	*ST6GALNAC3*	within
	2	53,391,808	4.948564 × 10^−7^	*FANCG*	331
		55,005,118	2.572812 × 10^−7^	*LOC121818624*	26,374
		55,047,205	2.521822 × 10^−7^	*ENSOARG00020031756*	within
CF	12	18,454,843	2.542981 × 10^−8^	*USH2A*	within
		72,689,402	6.228683 × 10^−8^	*SYT14*	within
	19	58,874,366	9.867142 × 10^−8^	*EFCC1*	within
>15%	1	15,993,581	5.558001 × 10^−7^	*EDN2*	32,531
		101,310,201	5.935847 × 10^−7^	*LOC114117513*	−1293
		103,394,621	4.247662 × 10^−7^	*ENSOARG00020030884*	within
		121,546,311	2.677888 × 10^−7^	*RCAN1*	within
		121,608,994	8.232058 × 10^−7^	*RCAN1*	within
		122,856,396	9.882277 × 10^−7^	*IFNAR1*	within
		229,038,319	3.510156 × 10^−7^	*ENSOARG00020038272*	within
		265,955,164	3.546715 × 10^−7^	*COL18A1*	within
	2	25,569,486	4.394279 × 10^−7^	*DIRAS2*	68,469
		16,8452,761	7.346249 × 10^−7^	*LRP1B*	within
		242,826,705	3.103179 × 10^−7^	*HMGCL*	5056
	4	11,699,947	3.954987 × 10^−7^	*CALCR*	−28,368
		121,492,684	8.785249 × 10^−7^	*LOC114114472*	−40,923
	5	31,265,184	3.916315 × 10^−7^	*ENSOARG00020027327*	within
		89,990,179	3.08966 × 10^−7^	*TRNAW-CCA*	−279,579
	6	117,917,615	3.670075 × 10^−7^	*CTBP1*	−899
	10	72,536,458	3.864163 × 10^−7^	*UGGT2*	within
	17	71,252,665	3.322203 × 10^−7^	*LOC105602956*	within
	18	22,794,738	7.483516 × 10^−7^	*LOC114118854*	within
		66,955,441	2.417579 × 10^−7^	*SIVA1*	within
	21	16,607	3.724726 × 10^−7^	*PANX1*	177,023
	26	2,331,482	3.70147 × 10^−7^	*CSMD1*	within
	X	21,855,901	3.363513 × 10^−7^	*PTCHD1*	−10,474
SL	1	2,983,391	3.575274 × 10^−7^	*TRAF3IP1*	within
		97,859,378	4.074127 × 10^−7^	*LOC114112215*	9239
		194,406,396	4.074127 × 10^−7^	*LOC121816689*	16,194
		195,679,749	4.082481 × 10^−7^	*FGF12*	within
		196,441,630	5.059276 × 10^−7^	*ENSOARG00020027852*	within
		198,957,601	7.336839 × 10^−7^	*TPRG1*	within
		227,004,375	1.747904 × 10^−7^	*NMD3*	within
		231,145,470	4.074127 × 10^−7^	*VEPH1*	within
	2	115,507,582	3.980196 × 10^−7^	*LOC106990902*	−20,393
		135,022,009	7.476657 × 10^−8^	*ENSOARG00020033964*	within
		135,336,861	5.380042 × 10^−7^	*CHRNA1*	within
	3	18,857,597	4.179918 × 10^−7^	*ADAM17*	within
		179,239,910	3.971237 × 10^−7^	*HMOX1, ENSOARG00020039035*	within
	4	43,547	4.107503 × 10^−7^	*LOC105605926*	265,080
		11,316,451	3.296337 × 10^−7^	*HEPACAM2*	within
		92,353,566	3.143381 × 10^−7^	*GRM8*	within
		93,899,832	3.592256 × 10^−7^	*SND1*	within
		93,938,145	5.156819 × 10^−7^	*LRRC4*	within
	8	13,764,777	1.733298 × 10^−7^	*NKAIN2*	within
		16,549,119	4.012221 × 10^−7^	*LOC132660198*	102,411
	11	14,111,738	3.757219 × 10^−7^	*ENSOARG00020021921*	within
	12	192,483	4.012221 × 10^−7^	*ENSOARG00020035247*	within
	17	37,935	4.012221 × 10^−7^	*LOC105605790*	243,265
	18	221,140	4.012221 × 10^−7^	*MKRN3*	303,877
		226,967	7.148295 × 10^−7^	*MKRN3*	298,050
	19	79,786	4.012221 × 10^−7^	*MRPS24*	142,918
	20	49,080,151	1.000487 × 10^−7^	*LOC114109591*	within
	21	6,275,544	4.693387 × 10^−7^	*GRM5, ENSOARG00020035270*	within
		40,000,782	4.009804 × 10^−7^	*TIGD3*	within
	23	48,181,894	4.356746 × 10^−7^	*ZBTB7C*	within
	X	109,190,351	4.158501 × 10^−7^	*TENM1*	within
		125,810,956	3.979989 × 10^−7^	*GUCY2F*	within
		127,195,428	4.009804 × 10^−7^	*VSIG1*	within
		127,703,493	4.928113 × 10^−7^	*FRMPD3*	within
		141,116,984	4.336624 × 10^−7^	*TRNAC-ACA*	−21,661
CRV	1	255,629,888	1.540812 × 10^−7^	*AMOTL2*	−6948
	2	191,763,534	9.518238 × 10^−7^	*LOC101116805*	112,736
	11	34,467,404	3.054953 × 10^−7^	*ENSOARG00020039760*	within

Trait: FD (fiber diameter), SD (standard deviation), CV% (coefficient of variation), CF% (comfort factor), <15% (percent of fibers under 15 microns), SL (staple length), CRV (fiber curvature). Chromosome (Chr) and position (Pos) of the SNP according to the reference genome GCA_016772045.1_ARS-UI_Ramb_v2.0. Distance (bp): distance in base pairs from the nearest gene to the associated SNPs (within if the SNPs is inside a gene, negative if the gene is downstream from the SNP, and positive if the gene is upstream from the SNP).

## Data Availability

All data contained within the article.
